# Supplementary figures and images for: Comparative structural dynamic analysis of GTPases
Source: PLoS Comput Biol. 2018 Nov 9;14(11):e1006364. doi: 10.1371/journal.pcbi.1006364 (PMC6249014; doi:10.1371/journal.pcbi.1006364)

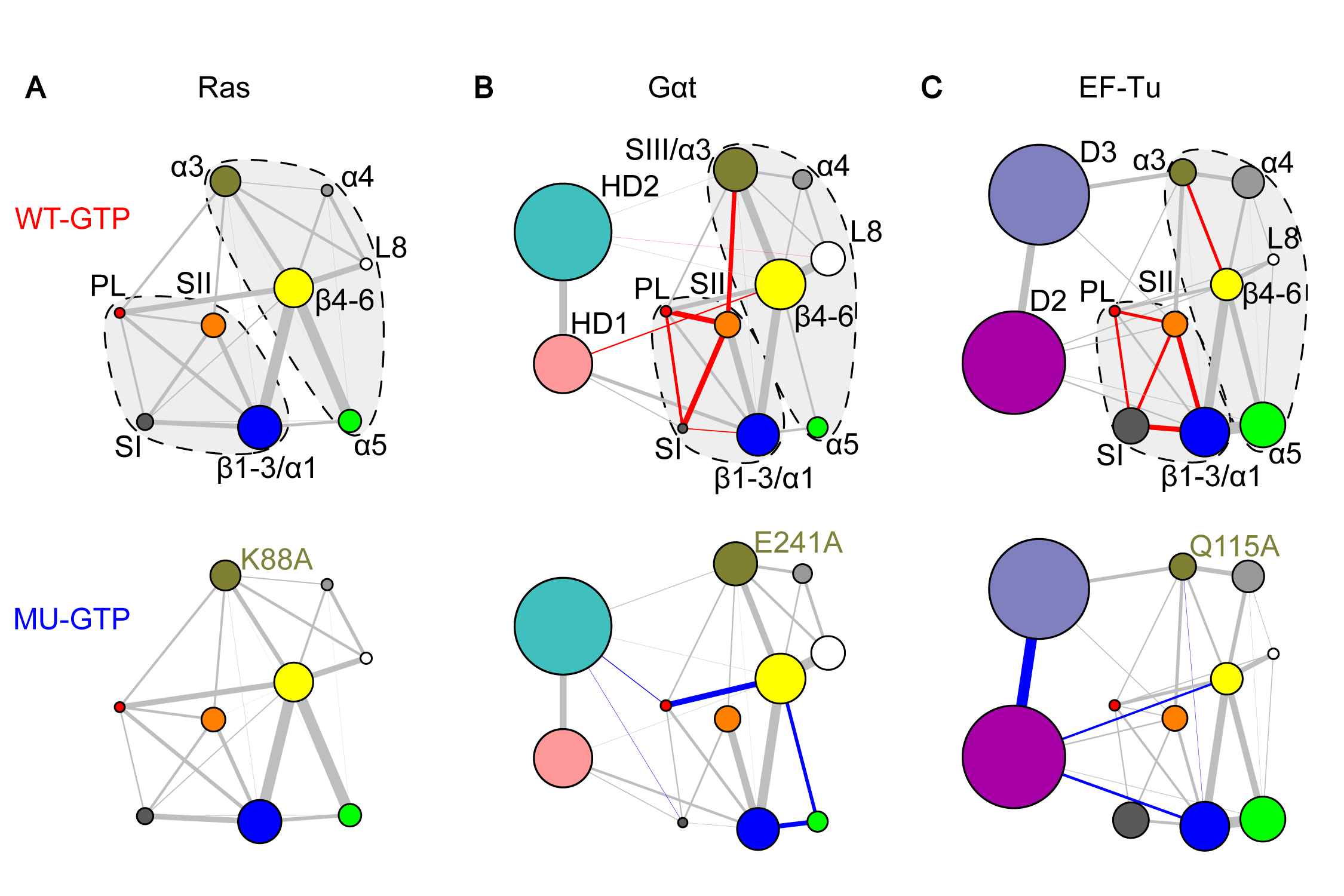

Supplement: S1 Fig — In each panel, networks of wild type GTP-bound (WT-GTP, top) and mutant GTP-bound (MU-GTP, bottom) are compared. Red and blue edges indicate enhanced WT or MU couplings that are significantly (p-value <0.05). All other lines are colored gray. Specific mutations E241AGαt (B) and Q115AEF-Tu (C) in α3 dramatically reduce the couplings between the functional regions PL, SI and SII, whereas the counterpart mutation K88ARas (A) has minor effects. (TIF) [file pcbi.1006364.s001.tif]

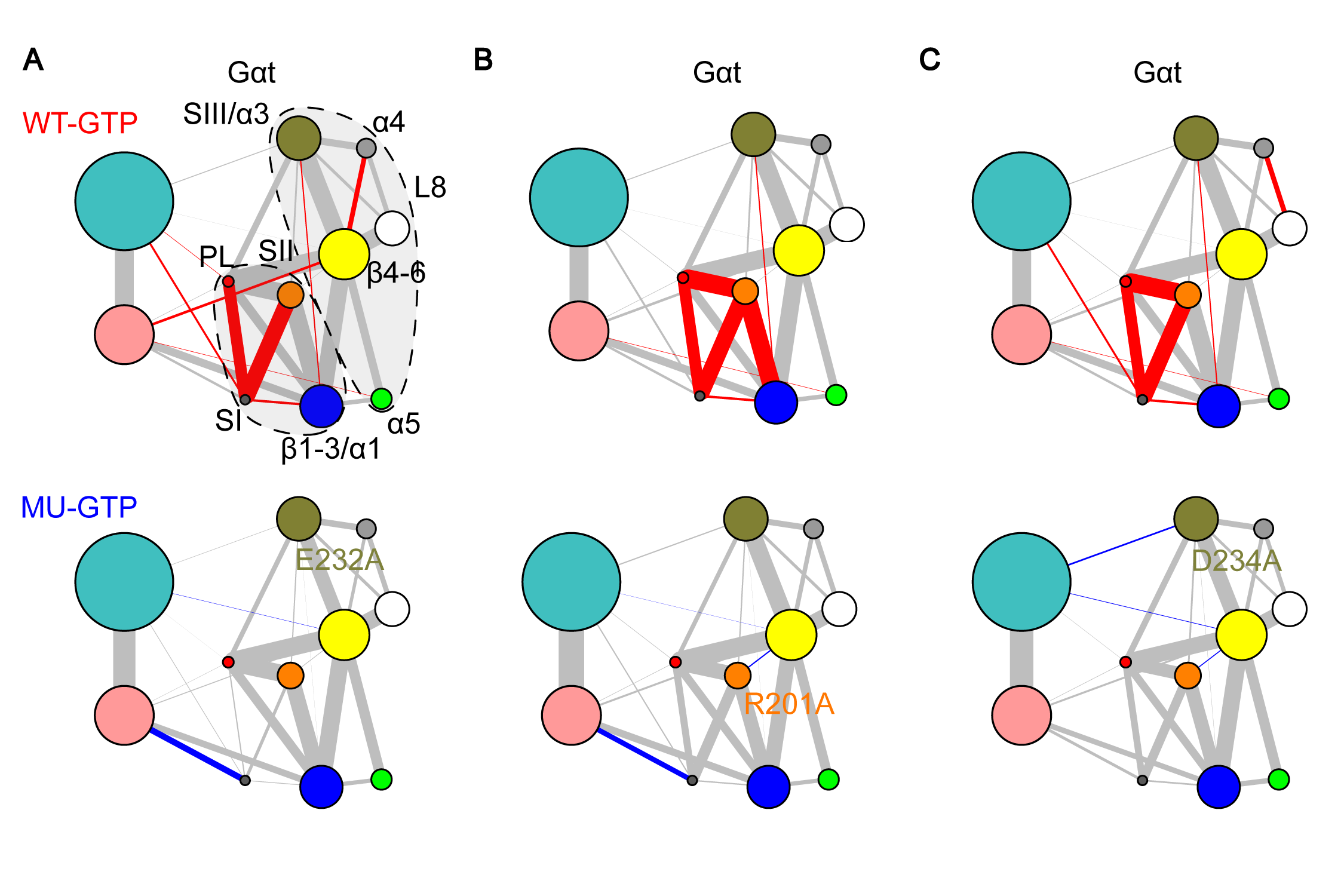

Supplement: S2 Fig — In each panel, networks of wild type GTP-bound (WT-GTP, top) and mutant GTP-bound (MU-GTP, bottom) are compared. Red and blue edges indicate enhanced WT or MU couplings that are significantly (p-value <0.05). All other lines are colored gray. Gαt specific mutations E232AGαt (A) in SIII dramatically reduce the couplings between the functional regions PL, SI and SII. Similar effects of mutations R201AGαt (B) and D234AGαt (C) are also observed in Gαt. (TIF) [file pcbi.1006364.s002.tif]

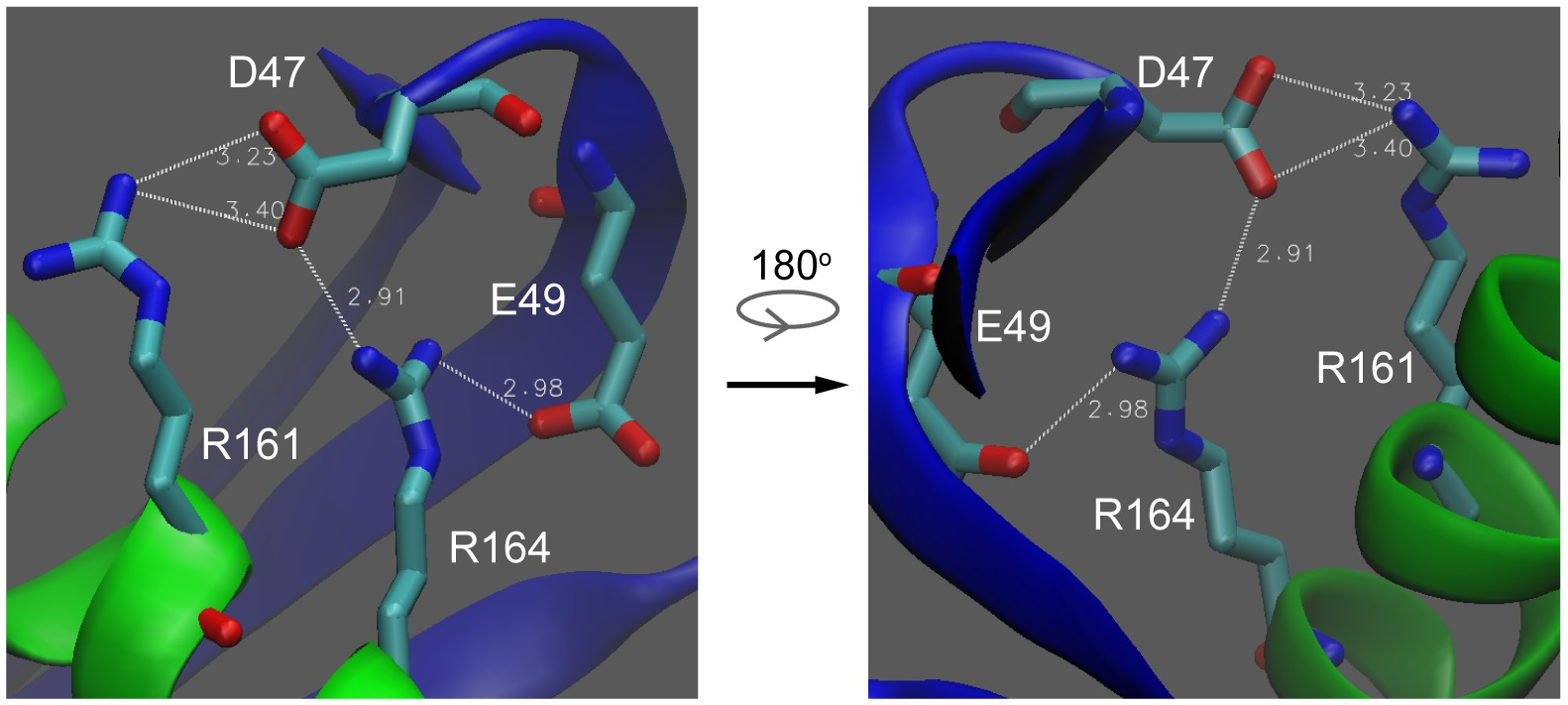

Supplement: S3 Fig — The L3 loop and helix α5 are shown as secondary structure cartoons in blue and green respectively. The side chains of the noted residues are highlighted, with oxygen atoms in red and nitrogen atoms in blue. Labeled distances are in the unit of Angstrom (Å). (TIF) [file pcbi.1006364.s003.tif]

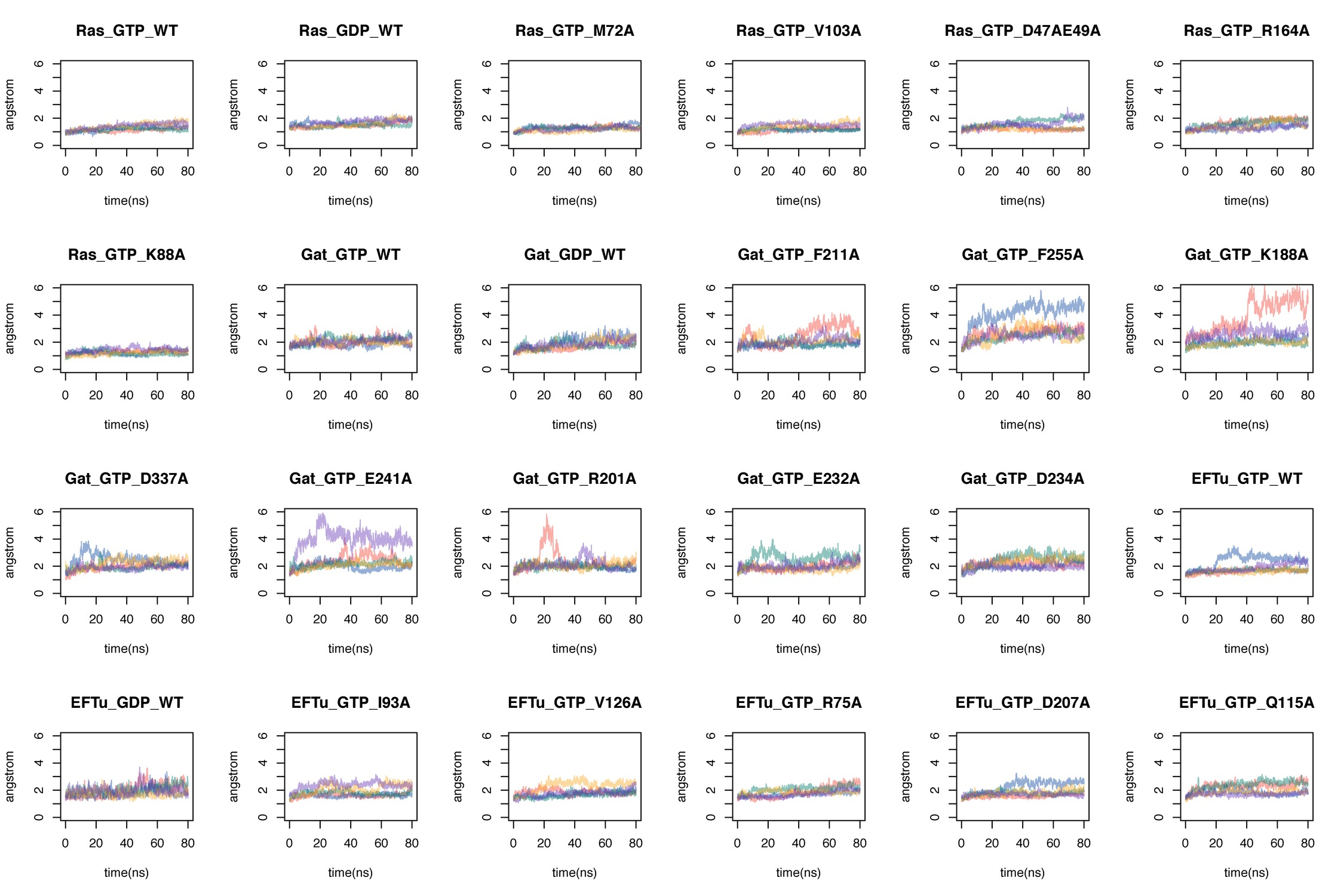

Supplement: S4 Fig — In each system, the five simulation replicates are shown in five different colors. (TIF) [file pcbi.1006364.s004.tif]
