# Supplementary material for: Comparative structural dynamic analysis of GTPases
Source: PLoS Comput Biol. 2018 Nov 9;14(11):e1006364. doi: 10.1371/journal.pcbi.1006364 (PMC6249014; doi:10.1371/journal.pcbi.1006364)
Supplement: S1 Table — (DOCX) [file pcbi.1006364.s005.docx]

**Supporting Information: S1 Table**

**Comparative structural dynamic analysis of GTPases**

Hongyang Li ^1^, Xin-Qiu Yao ^2^, Barry J. Grant ^3, *^

**^1^** Department of Computational Medicine and Bioinformatics, University of Michigan, 100 Washtenaw Avenue, Ann Arbor, MI 48109, USA.

**^2^** Department of Chemistry, Georgia State University, Atlanta, GA 30302-3965, USA.

**^3^** Division of Biological Sciences, Section of Molecular Biology, University of California, San Diego, La Jolla, CA 92093, USA.

* Corresponding author: [bjgrant@ucsd.edu](mailto:bjgrant@ucsd.edu)

**S1 Table. Analyzed crystallographic structures of Ras**

| PDB ID | Chain | Ligand |
| --- | --- | --- |
| 121P | A | GCP,MG |
| 1AGP | A | GNP,MG |
| 1BKD | R |  |
| 1BKD | S |  |
| 1CTQ | A | GNP,MG |
| 1GNP | A | AGN,MG |
| 1GNQ | A | CAG,MG |
| 1GNR | A | CAG,MG |
| 1HE8 | A |  |
| 1HE8 | B |  |
| 1JAH | A | GCP,MG |
| 1JAI | A | GCP,MN |
| 1K8R | A | GNP,MG |
| 1K8R | B |  |
| 1LF0 | A | CA,GNP,MG |
| 1LF5 | A | GDP,MG |
| 1LFD | A |  |
| 1LFD | B | GNP,MG |
| 1LFD | C |  |
| 1LFD | D | GNP,MG |
| 1NVU | Q | GTP,MG,PO4 |
| 1NVU | R | PO4 |
| 1NVU | S | PO4 |
| 1NVV | Q | GNP,MG,PO4 |
| 1NVV | R | PO4 |
| 1NVV | S | PO4 |
| 1NVW | Q | GNP,MG,PO4 |
| 1NVW | R | PO4 |
| 1NVW | S | PO4 |
| 1NVX | Q | GTP,MG |
| 1NVX | R | PO4 |
| 1NVX | S |  |
| 1P2S | A | ETF,GNP,MG |
| 1P2V | A | GNP,HEZ,MG |
| 1Q21 | A | GDP,MG |
| 1QRA | A | GTP,MG |
| 1WQ1 | G |  |
| 1WQ1 | R | AF3,GDP,MG |
| 1XCM | A | CSO,GNP,MG |
| 1XD2 | A | GDP,MG,PO4 |
| 1XD2 | B | PO4 |
| 1XD2 | C | PO4 |
| 1XJ0 | A | CSX,GDP,MG |
| 1ZVQ | A | GDP,MG |
| 1ZW6 | A | CA,CSO,GNP,MG |
| 221P | A | GNP,MG |
| 2C5L | A | GOL,GTP,MG |
| 2C5L | B | GOL,GTP,MG |
| 2C5L | C | GOL |
| 2C5L | D |  |
| 2CE2 | X | GDP,MG,XY2 |
| 2CL0 | X | GNP,MG,TRS,XY2 |
| 2CL6 | X | CAG,MG,XY2 |
| 2CL7 | X | GTP,MG,XY2 |
| 2CLC | X | GTP,MG,XY2 |
| 2EVW | X | CAG,MG,XY2 |
| 2Q21 | A | GDP,MG |
| 2QUZ | A | GDP,MG |
| 2RGA | A | CA,GNP,MG |
| 2RGB | A | CA,GNP,MG |
| 2RGC | A | CA,GNP,MG |
| 2RGD | A | CA,GNP,MG |
| 2UZI | H | ZN |
| 2UZI | L |  |
| 2UZI | R | GTP,MG,ZN |
| 2VH5 | H | ZN |
| 2VH5 | L |  |
| 2VH5 | R | GTP,MG,ZN |
| 3DDC | A | GNP,MG |
| 3DDC | B |  |
| 3GFT | A | GNP,MG,UNX |
| 3GFT | B | GNP,MG |
| 3GFT | C | GNP,MG |
| 3GFT | D | GNP,MG |
| 3GFT | E | GNP,MG |
| 3GFT | F | CIT,GNP,MG |
| 3I3S | R | CA,GNP,MG |
| 3K8Y | A | ACT,CA,GNP,MG |
| 3KKN | A | GNP,MG |
| 3L8Y | A | CA,GNP,MG,YCN,ZN |
| 3L8Z | A | CA,GNP,MG |
| 3LBH | A | ACT,CA,GNP,MG |
| 3LBI | A | ACT,CA,GNP,MG |
| 3LBN | A | CA,GNP,MG |
| 3OIU | A | ACT,CA,GNP,MG |
| 3OIW | A | ACT,CA,GNP,MG |
| 3RRY | A | CA,GNP,MG |
| 3RS0 | A | CA,GNP,MG,YEG |
| 3RS2 | A | CA,ETF,GNP,MG |
| 3RS3 | A | CA,GNP,HEX,MG |
| 3RS5 | A | CA,DMF,GNP,MG |
| 3RSO | A | CA,GNP,MG,RSG |
| 3V4F | A | CA,DTU,GNP,MG |
| 421P | A | GNP,MG |
| 4DLR | A | CA,DTU,GNP,MG |
| 4DLS | A | CA,GNP,MG |
| 4DLT | A | ACT,CA,GNP,MG |
| 4DLU | A | ACT,CA,GNP,MG |
| 4DLV | A | CA,DTT,GNP,MG |
| 4DLW | A | ACT,CA,GNP,MG |
| 4DLX | A | CA,DTU,GNP,MG |
| 4DLY | A | CA,DTT,GNP,MG |
| 4DLZ | A | CA,DTU,GNP,MG |
| 4DSN | A | EDO,GCP,MG |
| 4DSO | A | BEN,GOL,GSP,MG |
| 4DST | A | 9LI,ACT,DMS,EDO,GCP,GOL,MG |
| 4EFL | A | GNP,MG |
| 4EFM | A | GNP,MG |
| 4EFN | A | GNP,MG |
| 4EPR | A | GDP,MG |
| 4EPT | A | 0QW,GDP,MG |
| 4EPV | A | 0QX,GDP,MG |
| 4EPW | A | 0QV,GDP,MG |
| 4EPX | A | 0QR,GDP,MG |
| 4EPY | A | 0QY,GDP,MG |
| 4G3X | A | GNP,MG |
| 4G3X | B |  |
| 4K81 | A | GOL |
| 4K81 | B | GOL,GTP,MG |
| 4K81 | C | GOL |
| 4K81 | D | GTP,MG |
| 4K81 | E | GOL |
| 4K81 | F | GTP,MG |
| 4K81 | G |  |
| 4K81 | H | GTP,MG |
| 4L9W | A | CA,GNP,MG |
| 4LDJ | A | GDP,MG |
| 4LPK | A | CA,GDP |
| 4LPK | B | CA,GDP |
| 4LUC | A | 20G,CA,GDP |
| 4LUC | B | 20G,CA,GDP |
| 4LV6 | A | 20H,CA,GDP |
| 4LV6 | B | 20H,CA,GDP |
| 4NMM | A | MG,Y9Z |
| 4NYI | Q | GNP,MG |
| 4NYI | R |  |
| 4NYI | S | 2PX |
| 4NYJ | Q | GNP,MG |
| 4NYJ | R |  |
| 4NYJ | S | 2PZ |
| 4NYM | Q | GNP,MG |
| 4NYM | R |  |
| 4NYM | S | RND |
| 4OBE | A | GDP,MG |
| 4OBE | B | GDP,MG |
| 4PZY | A | GDP,MG |
| 4PZY | B | 2XR,GDP,MG |
| 4PZZ | A | 2XO,GDP,MG |
| 4Q21 | A | GDP,MG |
| 4QL3 | A | GDP,MG |
| 4TQ9 | A | GDP,MG |
| 4TQ9 | B | GDP,MG |
| 4TQA | A | GDP,MG |
| 4TQA | B | GDP,MG |
| 4URU | R |  |
| 4URU | S | 6W2 |
| 4URV | R | FMT |
| 4URV | S | FMT,UMK |
| 4URW | R |  |
| 4URW | S | DXO |
| 4URX | R |  |
| 4URX | S | FK1,FMT,HXY |
| 4URY | R | RV1 |
| 4URY | S | RV1 |
| 4URZ | R | VJP |
| 4URZ | S |  |
| 4US0 | R | NEQ |
| 4US0 | S |  |
| 4US1 | R | L71 |
| 4US1 | S |  |
| 4US2 | R | L7S |
| 4US2 | S |  |
| 521P | A | GTP,MG |
| 5B2Z | A | CA,GNP,MG |
| 5B30 | A | CA,GNP,MG |
| 5F2E | A | 5UT,GDP,GLY,GOL,MG |
| 5P21 | A | GNP,MG |
| 621P | A | GNP,MG |
| 6Q21 | A | GCP,MG |
| 6Q21 | B | GCP,MG |
| 6Q21 | C | GCP,MG |
| 6Q21 | D | GCP,MG |
| 721P | A | GNP,MG |
| 821P | A | GNP,MG |
