# Supplementary material for: Comparative structural dynamic analysis of GTPases
Source: PLoS Comput Biol. 2018 Nov 9;14(11):e1006364. doi: 10.1371/journal.pcbi.1006364 (PMC6249014; doi:10.1371/journal.pcbi.1006364)
Supplement: S2 Table — (DOCX) [file pcbi.1006364.s006.docx]

**Supporting Information: S2 Table**

**Comparative structural dynamic analysis of GTPases**

Hongyang Li ^1^, Xin-Qiu Yao ^2^, Barry J. Grant ^3, *^

**^1^** Department of Computational Medicine and Bioinformatics, University of Michigan, 100 Washtenaw Avenue, Ann Arbor, MI 48109, USA.

**^2^** Department of Chemistry, Georgia State University, Atlanta, GA 30302-3965, USA.

**^3^** Division of Biological Sciences, Section of Molecular Biology, University of California, San Diego, La Jolla, CA 92093, USA.

* Corresponding author: [bjgrant@ucsd.edu](mailto:bjgrant@ucsd.edu)

**S21 Table. Analyzed crystallographic structures of G protein α subunit**

| PDB ID | Chain | Ligand |
| --- | --- | --- |
| 1TND | A | CAC,GSP,MG |
| 1TND | B | CAC,GSP,MG |
| 1TND | C | CAC,GSP,MG |
| 1TAD | A | ALF,CA,CAC,GDP |
| 1TAD | B | ALF,CA,CAC,GDP |
| 1TAD | C | ALF,CA,CAC,GDP |
| 1TAG | A | GDP,MG |
| 3V00 | C | GDP |
| 3V00 | B | GDP |
| 3V00 | A | GDP |
| 1FQJ | A | ALF,GDP,MG |
| 1FQJ | D | ALF,GDP,MG |
| 1FQK | A | ALF,GDP,MG |
| 1FQK | C | ALF,GDP,MG |
| 1GOT | A | MSE |
| 2XNS | A | GDP,SRT |
| 2XNS | B | GDP,SRT |
| 1KJY | A | CS,GDP |
| 1KJY | C | CS,GDP,MG |
| 2OM2 | A | GDP,MG |
| 2OM2 | C | GDP,MG |
| 4G5Q | A | GDP,SO4 |
| 4G5Q | D | CIT,GDP,SO4 |
| 1GP2 | A | GDP |
| 1AGR | A | ALF,CIT,GDP,MG |
| 1AGR | D | ALF,CIT,GDP,MG |
| 1CIP | A | GNP,MG |
| 1GFI | A | ALF,GDP,MG |
| 1GIA | A | GSP,MG |
| 2GTP | B | ALF,GDP,MG |
| 2ZJY | A | ALF,GDP,MG |
| 3ONW | A | GDP,SO4 |
| 3ONW | B | GDP,SO4 |
| 1BH2 | A | GSP,MG |
| 1GG2 | A | GDP |
| 1GIT | A | GDP,PO4 |
| 3QI2 | A | GDP,GOL,SO4 |
| 3QI2 | B | GDP,SO4 |
| 1SVK | A | ALF,GDP,MG |
| 1SVS | A | GNP,MG |
| 3FFA | A | GSP,MG,SO4 |
| 1GIL | A | GSP,MG |
| 1AS0 | A | GSP,MG,SO4 |
| 1AS2 | A | GDP,PO4 |
| 3QE0 | B | GDP,MG |
| 2ODE | A | ALF,GDP,MG |
| 2ODE | C | ALF,GDP,MG |
| 2V4Z | A | ALF,GDP,MG |
| 4G5R | A | GDP,SO4 |
| 2IHB | A | ALF,GDP,MG |
| 4G5O | A | CIT,GDP,SO4 |
| 4G5O | D | CIT,GDP,SO4 |
| 3C7K | C | ALF,GDP,MG |
