# Supplementary material for: Comparative structural dynamic analysis of GTPases
Source: PLoS Comput Biol. 2018 Nov 9;14(11):e1006364. doi: 10.1371/journal.pcbi.1006364 (PMC6249014; doi:10.1371/journal.pcbi.1006364)
Supplement: S3 Table — (DOCX) [file pcbi.1006364.s007.docx]

**Supporting Information: S3 Table**

**Comparative structural dynamic analysis of GTPases**

Hongyang Li ^1^, Xin-Qiu Yao ^2^, Barry J. Grant ^3, *^

**^1^** Department of Computational Medicine and Bioinformatics, University of Michigan, 100 Washtenaw Avenue, Ann Arbor, MI 48109, USA.

**^2^** Department of Chemistry, Georgia State University, Atlanta, GA 30302-3965, USA.

**^3^** Division of Biological Sciences, Section of Molecular Biology, University of California, San Diego, La Jolla, CA 92093, USA.

* Corresponding author: [bjgrant@ucsd.edu](mailto:bjgrant@ucsd.edu)

**S3 Table. Analyzed crystallographic structures of EF-Tu**

| PDB ID | Chain | Ligand |
| --- | --- | --- |
| 1B23 | P | GNP,MG,SO4 |
| 1B23 | R | 4SU,5MU,CYS,H2U,MG,MIA,PSU |
| 1D8T | A | ACT,GDP,MG |
| 1D8T | B | ACT,GDP,MG |
| 1D8T | C | BB6,BB7,BB8,BB9,MEN,MH6,NH2 |
| 1D8T | D | BB6,BB7,BB8,BB9,MEN,MH6,NH2 |
| 1DG1 | G | GDP,MG |
| 1DG1 | H | GDP,MG |
| 1EFC | A | GDP,MG |
| 1EFC | B | GDP,MG |
| 1EFT | A | GNP,MG |
| 1EXM | A | GNP,MG |
| 1OB2 | A | GNP,KIR,MG,SUC |
| 1OB2 | B | 1MA,2MG,5MC,7MG,H2U,M2G,OMC,OMG,PHA,PSU,YG |
| 1OB5 | A | ENX,GNP,MG |
| 1OB5 | B | 1MA,2MG,5MC,5MU,7MG,H2U,M2G,OMC,OMG,PHA,PSU,YG |
| 1OB5 | C | ENX,GNP,MG |
| 1OB5 | D | 1MA,2MG,5MC,5MU,7MG,H2U,M2G,OMC,OMG,PHA,PSU,YG |
| 1OB5 | E | ENX,GNP,MG |
| 1OB5 | F | 1MA,2MG,5MC,5MU,7MG,H2U,M2G,OMC,OMG,PHA,PSU,YG |
| 1TTT | A | GNP,MG |
| 1TTT | B | GNP,MG |
| 1TTT | C | GNP,MG |
| 1TTT | D | 1MA,2MG,5MC,5MU,7MG,H2U,M2G,MG,OMC,OMG,PHE,PSU,YYG |
| 1TTT | E | 1MA,2MG,5MC,5MU,7MG,H2U,M2G,MG,OMC,OMG,PHE,PSU,YYG |
| 1TTT | F | 1MA,2MG,5MC,5MU,7MG,H2U,M2G,MG,OMC,OMG,PHE,PSU,YYG |
| 1TUI | A | GDP,MG |
| 1TUI | B | GDP,MG |
| 1TUI | C | GDP,MG |
| 2C77 | A | GNP,MG,PEG |
| 2C77 | B | BB6,BB7,BB8,BB9,MEN,MH6,NH2 |
| 2C78 | A | GNP,MG,PUL |
| 2FX3 | A | GDP,MG |
| 3U6K | A | GDP,MG |
| 3U6K | B | GDP,MG |
| 3U6K | C | 9BB,BB6,BB7,BB8,BB9,MEN,MH6 |
| 3U6K | D | 9BB,BB6,BB7,BB8,BB9,MEN,MH6 |
| 4G5G | A | GDP,MG,SO4 |
| 4G5G | I | 05N,BB6,BB9,H14,MH6,NH2 |
| 4H9G | A | 14J,GNP,MG,NH4,SO4 |
| 4LBV | A | CL,GNP,MG,NH4,SO4 |
| 4LBW | A | GNP,MG,NH4,SO4 |
| 4LBY | A | GNP,MG,NH4,SO4 |
| 4LBZ | A | GNP,MG,NH4,SO4 |
| 4LC0 | A | GNP,MG,NH4,SO4 |
| 4P3Y | A | GDP,GOL,MG |
| 4P3Y | B |  |
| 4ZV4 | A | GDP,MG |
| 4ZV4 | B | GDP,MG |
| 4ZV4 | C |  |
| 4ZV4 | D |  |
