# Supplementary material for: Comparative structural dynamic analysis of GTPases
Source: PLoS Comput Biol. 2018 Nov 9;14(11):e1006364. doi: 10.1371/journal.pcbi.1006364 (PMC6249014; doi:10.1371/journal.pcbi.1006364)
Supplement: S4 Table — The numbers represent the residue-wise contributions to inter-community couplings. For example, the sum of correlations between residue M72 in SII and all residues in SIII/ α3 is 1.19 (after filtering by contact map). The first row contains common counterpart residues (red) connecting SII and SIII/α3 in three proteins. The second row contains family-specific functional residues: residues in Gαt and EF-Tu (blue) contribute to the dynamic correlations between SII and SIII/α3, whereas their counterparts in Ras (green) have no contributions. The third row contains Gαt specific residue in SIII, which has no counterparts in the other two proteins. (DOCX) [file pcbi.1006364.s008.docx]

**Supporting Information: S4 Table**

**Comparative structural dynamic analysis of GTPases**

Hongyang Li ^1^, Xin-Qiu Yao ^2^, Barry J. Grant ^3, *^

**^1^** Department of Computational Medicine and Bioinformatics, University of Michigan, 100 Washtenaw Avenue, Ann Arbor, MI 48109, USA.

**^2^** Department of Chemistry, Georgia State University, Atlanta, GA 30302-3965, USA.

**^3^** Division of Biological Sciences, Section of Molecular Biology, University of California, San Diego, La Jolla, CA 92093, USA.

* Corresponding author: [bjgrant@ucsd.edu](mailto:bjgrant@ucsd.edu)

**S4 Table. Residue-wise contributions to inter-community couplings.**

The numbers represent the residue-wise contributions to inter-community couplings. For example, the sum of correlations between residue M72 in SII and all residues in SIII/ α3 is 1.19 (after filtering by contact map). The first row contains common counterpart residues (red) connecting SII and SIII/α3 in three proteins. The second row contains family-specific functional residues: residues in Gαt and EF-Tu (blue) contribute to the dynamic correlations between SII and SIII/α3, whereas their counterparts in Ras (green) have no contributions. The third row contains Gαt specific residue in SIII, which has no counterparts in the other two proteins.

| SII | | | SIII/α3 | | |
| --- | --- | --- | --- | --- | --- |
| Ras | Gαt | EF-Tu | Ras | Gαt | EF-Tu |
| M72 | F211 | I93 | V103 | F255 | V126 |
| 1.19 | 0.5 | 0.88 | 0.96 | 0.26 | 1.71 |
| E62 | R201 | A86 | K88 | E241 | Q115 |
| 0 | 1.63 | 0.23 | 0 | 1.14 | 0.23 |
|  |  |  | NA | E232(SIII) | NA |
|  |  |  |  | 1.03 |  |
