# Supplementary material for: Comparative structural dynamic analysis of GTPases
Source: PLoS Comput Biol. 2018 Nov 9;14(11):e1006364. doi: 10.1371/journal.pcbi.1006364 (PMC6249014; doi:10.1371/journal.pcbi.1006364)
Supplement: S5 Table — (DOCX) [file pcbi.1006364.s009.docx]

**Supporting Information: S5 Table**

**Comparative structural dynamic analysis of GTPases**

Hongyang Li ^1^, Xin-Qiu Yao ^2^, Barry J. Grant ^3, *^

**^1^** Department of Computational Medicine and Bioinformatics, University of Michigan, 100 Washtenaw Avenue, Ann Arbor, MI 48109, USA.

**^2^** Department of Chemistry, Georgia State University, Atlanta, GA 30302-3965, USA.

**^3^** Division of Biological Sciences, Section of Molecular Biology, University of California, San Diego, La Jolla, CA 92093, USA.

* Corresponding author: [bjgrant@ucsd.edu](mailto:bjgrant@ucsd.edu)

**S5 Table: Summary of systems simulated.**

| **Protein** | **Nucleotide** | **Mutation** | **Simulation Length** |
| --- | --- | --- | --- |
| Ras | GTP | WT | 80 ns |
| Ras | GDP | WT | 80 ns |
| Ras | GTP | M72A | 80 ns |
| Ras | GTP | V103A | 80 ns |
| Ras | GTP | D47A; E49A | 80 ns |
| Ras | GTP | R164A | 80 ns |
| Ras | GTP | K88A | 80 ns |
| Gαt | GTP | WT | 80 ns |
| Gαt | GDP | WT | 80 ns |
| Gαt | GTP | F211A | 80 ns |
| Gαt | GTP | F255A | 80 ns |
| Gαt | GTP | K188A | 80 ns |
| Gαt | GTP | D337A | 80 ns |
| Gαt | GTP | E241A | 80 ns |
| Gαt | GTP | R201A | 80 ns |
| Gαt | GTP | E232A | 80 ns |
| Gαt | GTP | D234A | 80 ns |
| EF-Tu | GTP | WT | 80 ns |
| EF-Tu | GDP | WT | 80 ns |
| EF-Tu | GTP | I93A | 80 ns |
| EF-Tu | GTP | V126A | 80 ns |
| EF-Tu | GTP | R75A | 80 ns |
| EF-Tu | GTP | D207A | 80 ns |
| EF-Tu | GTP | Q115A | 80 ns |
| EF-Tu | GTP | D234A | 80 ns |
|  |  |  | total: 1920 ns |
